# STC2 promotes head and neck squamous cell carcinoma metastasis through modulating the PI3K/AKT/Snail signaling

**DOI:** 10.18632/oncotarget.13355

**Published:** 2016-11-15

**Authors:** Shuwen Yang, Qinghai Ji, Bin Chang, Yan Wang, Yongxue Zhu, Duanshu Li, Caiping Huang, Yulong Wang, Guohua Sun, Ling Zhang, Qing Guan, Jun Xiang, Wenjun Wei, Zhongwu Lu, Tian Liao, Jiao Meng, Ziliang Wang, Ben Ma, Li Zhou, Yu Wang, Gong Yang

**Affiliations:** ^1^ Department of Head & Neck Surgery, Fudan University Shanghai Cancer Center, Shanghai 200032, China; ^2^ Department of Oncology, Shanghai Medical College, Fudan University, Shanghai 200032, China; ^3^ Cancer Research Institute, Fudan University Shanghai Cancer Center, Shanghai 200032, China; ^4^ Department of Pathology, Fudan University Shanghai Cancer Center, Shanghai 200032, China; ^5^ Central Laboratory, The Fifth People's Hospital of Shanghai, Fudan University, Shanghai 200240, China

**Keywords:** STC2, pAKT, Snail, HNSCC, metastasis

## Abstract

The mammalian peptide hormone stanniocalcin 2 (STC2) plays an oncogenic role in many human cancers. However, the exact function of STC2 in human head and neck squamous cell carcinoma (HNSCC) is unclear. We aimed to examine the function and clinical significance of STC2 in HNSCC. Using in vitro and in vivo assays, we show that overexpression of STC2 suppressed cell apoptosis, promoted cell proliferation, migration, invasion, and cell cycle arrest at the G1/S transition. By contrast, silencing of STC2 inhibited these activities. We further show that STC2 upregulated the phosphorylation of AKT and enhanced HNSCC metastasis via Snail-mediated increase of vimentin and decrease of E-cadherin. These responses were blocked by silencing of STC2/Snail expression or inhibition of pAKT activity. Furthermore, clinical data indicate that high STC2 expression was associated with high levels of pAKT and Snail in tumor samples from HNSCC patients with regional lymph node metastasis (P < 0.01). Thus, we conclude that STC2 controls HNSCC metastasis via the PI3K/AKT/Snail signaling axis and that targeted therapy against STC2 may be a novel strategy to effectively treat patients with metastatic HNSCC.

## INTRODUCTION

Primary head and neck squamous cell carcinoma (HNSCC), which includes cancers of the oral cavity, nasopharynx, oropharynx, and larynx, is the sixth most common cancer worldwide and the most frequent malignancy of the upper aerodigestive tract [1]. The disease is characterized by the intricate anatomy of the primary tumor sites, locoregional spread, distant metastasis, recurrences, the presence of secondary primary tumors, and detection at late stages. The 5-year survival rate for patients with HNSCC is only 30%, which is mainly due to locoregional metastasis in more than 60% of patients [2]. In addition to experiencing poor survival rates, HNSCC patients also suffer more pain and have a poorer quality of life compared to other cancer patients [3]. Despite the identification of multiple HNSCC risk factors, as well as the use of more aggressive surgical treatments, radiotherapy, and chemotherapy, the prognosis and survival rate of this disease have not improved significantly over the last few decades [4]. Therefore, it is vital to explore new therapeutic targets in order to increase patient survival and to prevent tumor dissemination.

STC2, a member of the stanniocalcin family, encodes a 302-amino acid protein and possesses high sequence homology to STC1. STC2 is expressed predominantly as a 2-kb transcript, although a 4.4-kb transcript has also been detected in some tissues. STC2 has been found to play important roles in many physiological processes such as bone development, reproduction, wound healing, angiogenesis and modulation of inflammatory responses [5, 6]. However, previous studies have also shown that STC2 is upregulated in human gastric cancers, neuroblastomas, breast cancers, colorectal cancers, and renal cell carcinomas [7–12]. Moreover, a recent study of 214 clinical samples suggests that STC2 protein expression may be a valuable biomarker for laryngeal squamous cell carcinoma malignancies, as well as a prognostic marker for poor outcome following surgery [13]. However, the precise role of STC2 in HNSCC is unclear.

In this study, we employed cell lines, animal models, and human specimens to investigate the effects of STC2 on HNSCC cell proliferation, cell cycle transition, invasion, and metastasis. Our data strongly indicate that STC2 may represent a novel and promising therapeutic target for HNSCC.

## RESULTS

### STC2 promotes HNSCC cell proliferation in vitro

**Figure 1:**
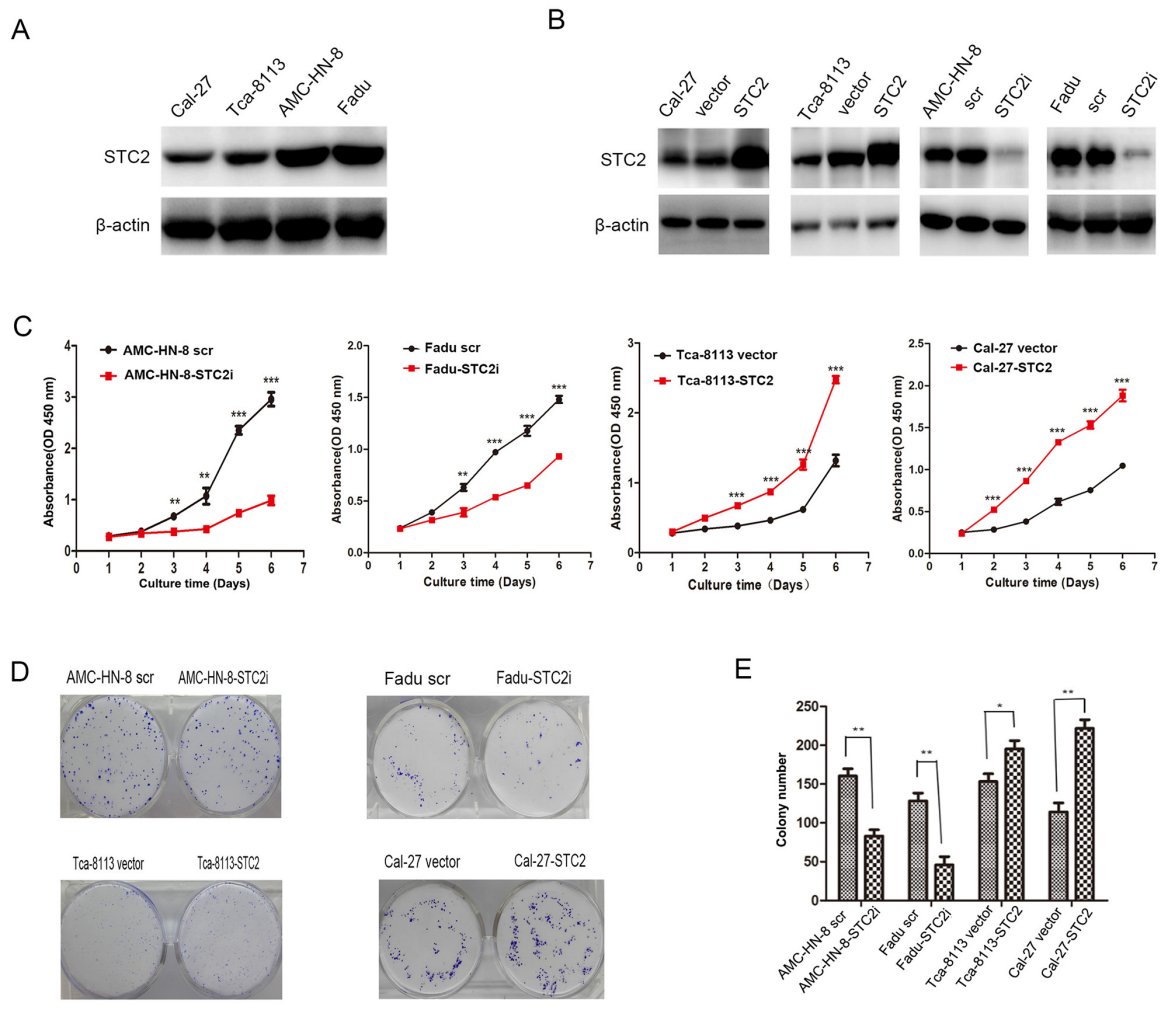
STC2 promotes HNSCC cell proliferation in vitro **A**. Detection of STC2 by Western blotting in HNSCC cell lines. **B**. Detection of STC2 silencing or overexpressing cell lines. **C**.The effects of STC2 on HNSCC cell proliferation. The cell numbers were determined at the indicated time intervals using Cell Counting Kit-8 reagent. Data are expressed as the mean +/− SD of five replicates. The experiments were repeated three times, and representative results are shown. **P < 0.01and***P < 0.001. **D, E**. STC2 expression enhanced the colony formation ability of HNSCC cells. The histogram represents the mean colony number of 3 independent experiments. *P< 0.05 and **P<0.01.

**Figure 2:**
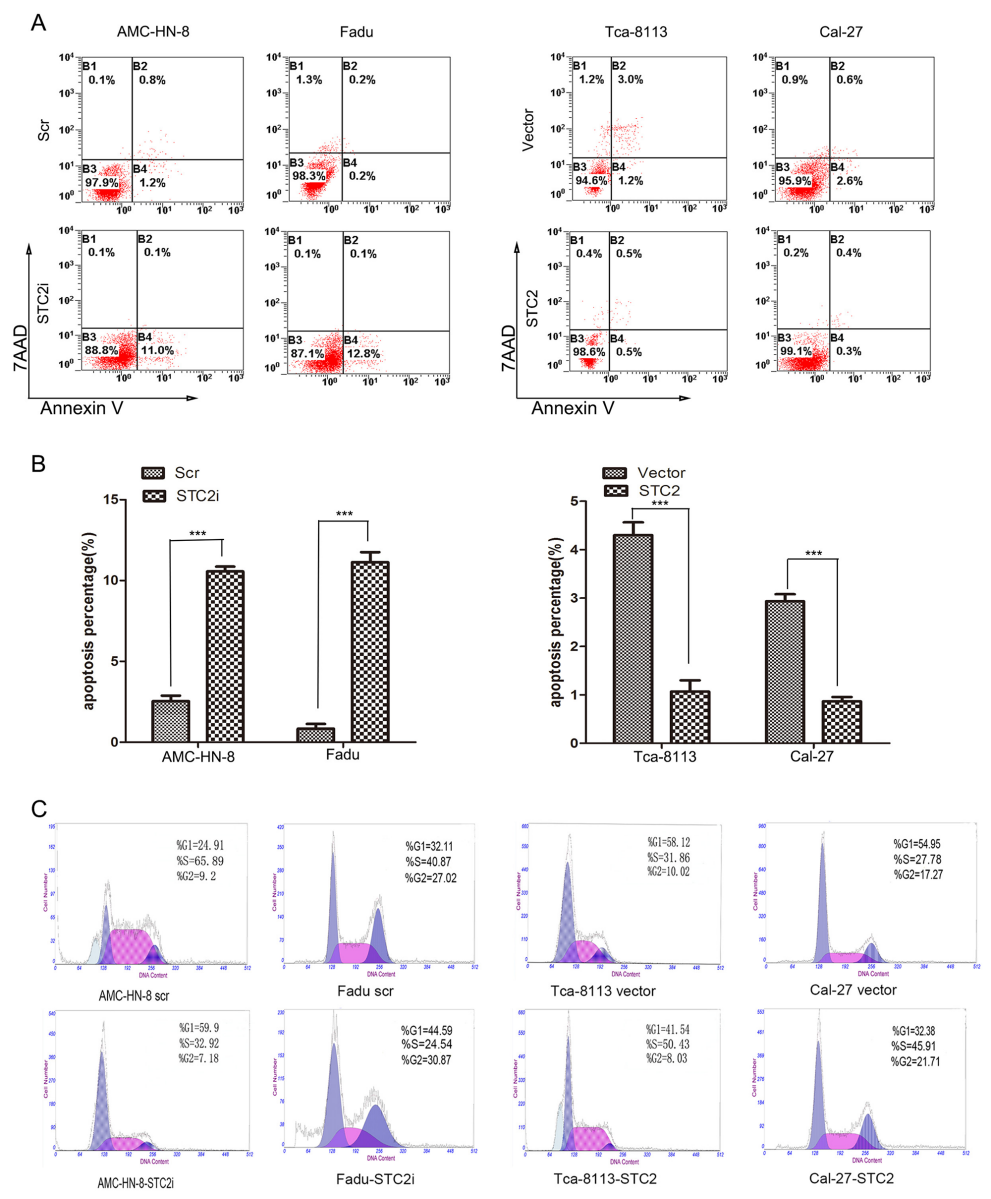
STC2 suppresses cell apoptosis **A, B**. After the same treatment, the cell apoptosis was analyzed by Annexin V/7AAD staining assay. The percentage of apoptotic cells is shown as the means ± SEM from three independent experiments. ***P< 0.001. **C**. Detection of cell cycle by flow cytometry analysis. The proportion of cells in G0/G1 phase was increased after transfection with STC2 shRNA and S phase was increased after transfection with STC2 cDNA.

Although some studies have shown that STC2 promotes cell proliferation [9, 11, 14], other studies have demonstrated that the expression of STC2 inhibits cell proliferation [5, 7, 15–17]. To investigate the role of STC2 in HNSCC cell proliferation, western blot analysis was first used to detect the level of STC2 expression in four HNSCC cell lines (Figure 1A). A high level of STC2 was detected in AMC-HN-8 and FaDu cells, while low levels of STC2 were detected in Cal-27 and Tca-8113 cells. We then transduced AMC-HN-8 and FaDu cells with a retroviral vector encoding a STC2 shRNA, and transfected STC2 cDNA into Cal-27 and Tca-8113 cells. Compared to control cells treated with an empty vector or a scrambled shRNA (scr), STC2 was markedly overexpressed or silenced in cells treated, respectively, with STC2 cDNA and STC2 shRNA (STC2i) (Figure 1B). We then tested the impact of STC2 on HNSCC cell proliferation *in vitro*. Using a CCK8 assay, we found that overexpression of STC2 promoted HNSCC cell proliferation, whereas downregulation of STC2 inhibited this effect (p<0.001, Figure 1C). Moreover in colony formation assay, wherein cells treated with STC2 shRNA (STC2i) generated fewer colonies in monolayer culture compared to controls, whereas cells expressing STC2 cDNA exhibited enhanced colony formation capacity (Figure 1D–1E). These data were consistent with Annexin V and 7AAD binding assay on apoptosis in HNSCC cells. As shown in Figure 2A and 2B, flow cytometry revealed that the amount of apoptotic cells in the cells transfected STC2 shRNA were greatly increased while in the cells transfected STC2 cDNA were decreased. The AMC-HN-8 cells group had apoptotic cells up to 11.1% of the total and 12.8% for the Fadu cells group. Flow cytometry analysis was then used to detect changes in the cell cycle distribution of HNSCC cells. The proportion of cells in G0/G1 phase was markedly increased after transfection with STC2 shRNA, whereas the proportion of cells in S phase was increased after transfection with STC2 cDNA (Figure 2C). Taken together, these results indicate that STC2 may exert a strong growth-permissive effect on HNSCC cells *in vitro*, possibly by regulating passage through the G1/S cell cycle transition.

### STC2 promotes HNSCC cell migration, invasion, and tumor metastasis

**Figure 3:**
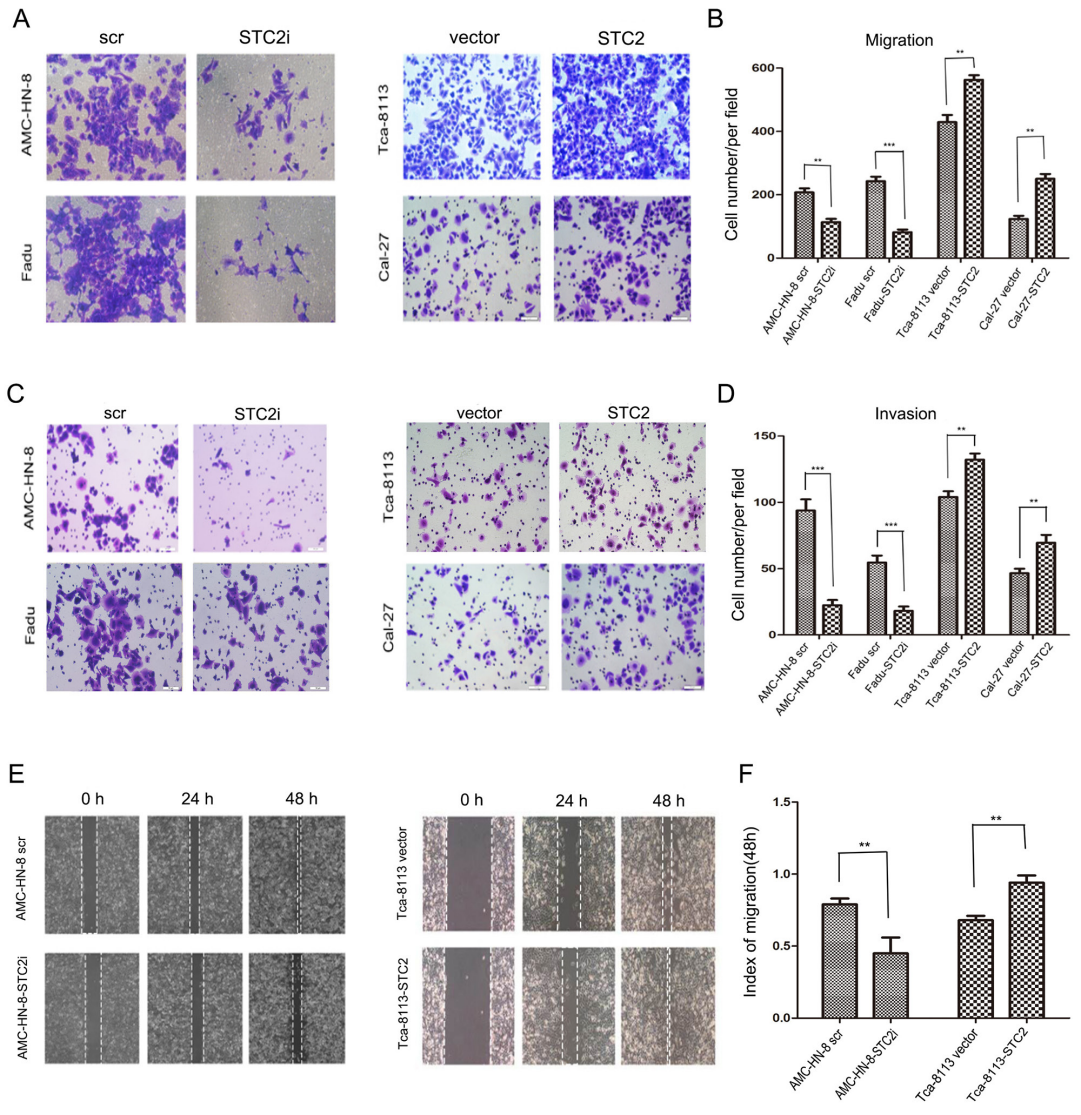
STC2 strengthens HNSCC cell migration, invasion, and tumor metastasis **A, B**. Detection of cell migration by using a high throughput screening multi-well insert 24-well two-chamber plates. Five images were taken randomly for each experiment, and each experiment was repeated three times. Magnification, ×200 (A). Quantitative analysis of the number of migrated cells. (**P < 0.01 and ***P < 0.001.) Error bars = 95%CIs (B). **C, D**. Representative images of the invasion assay. Magnification, × 200. (**P < 0.01and***P < 0.001). Error bars = 95%CIs. **E, F**. Detection of migration by scratching assay. Cells were incubated in 6-well plate over-night to yield monolayer confluence. Quantitative analysis of migration speed using migration index (P < 0.05). Error bars = 95% CIs.

We next assessed the role of STC2 in the migration and invasion of HNSCC cells using high-throughput 24-well plates containing polycarbonate membrane inserts. As shown in Figure 3A-3B, a greater number of Cal-27-STC2 and Tca-8113-STC2 cells migrated through the chamber membranes compared to their corresponding control cells, whereas fewer AMC-HN-8-STC2i and FaDu-STC2i cells migrated compared to their respective controls. Similar results were obtained for the invasion assay (Figure 3C–3D). In addition, we also used a scratch assay to assess the speed of cancer cell migration. As shown in Figure 3E-3F, after 24 h and 48 h of culture, the migration speed of cells treated with STC2 shRNA (STC2i) was much slower compared to control cells, whereas cells expressing STC2 cDNA more rapidly closed the scratched wound than did their respective controls. These results suggest that STC2 may enhance the invasive potential of HNSCC cells.

### STC2 promotes the growth of HNSCC *in vivo*

**Figure 4:**
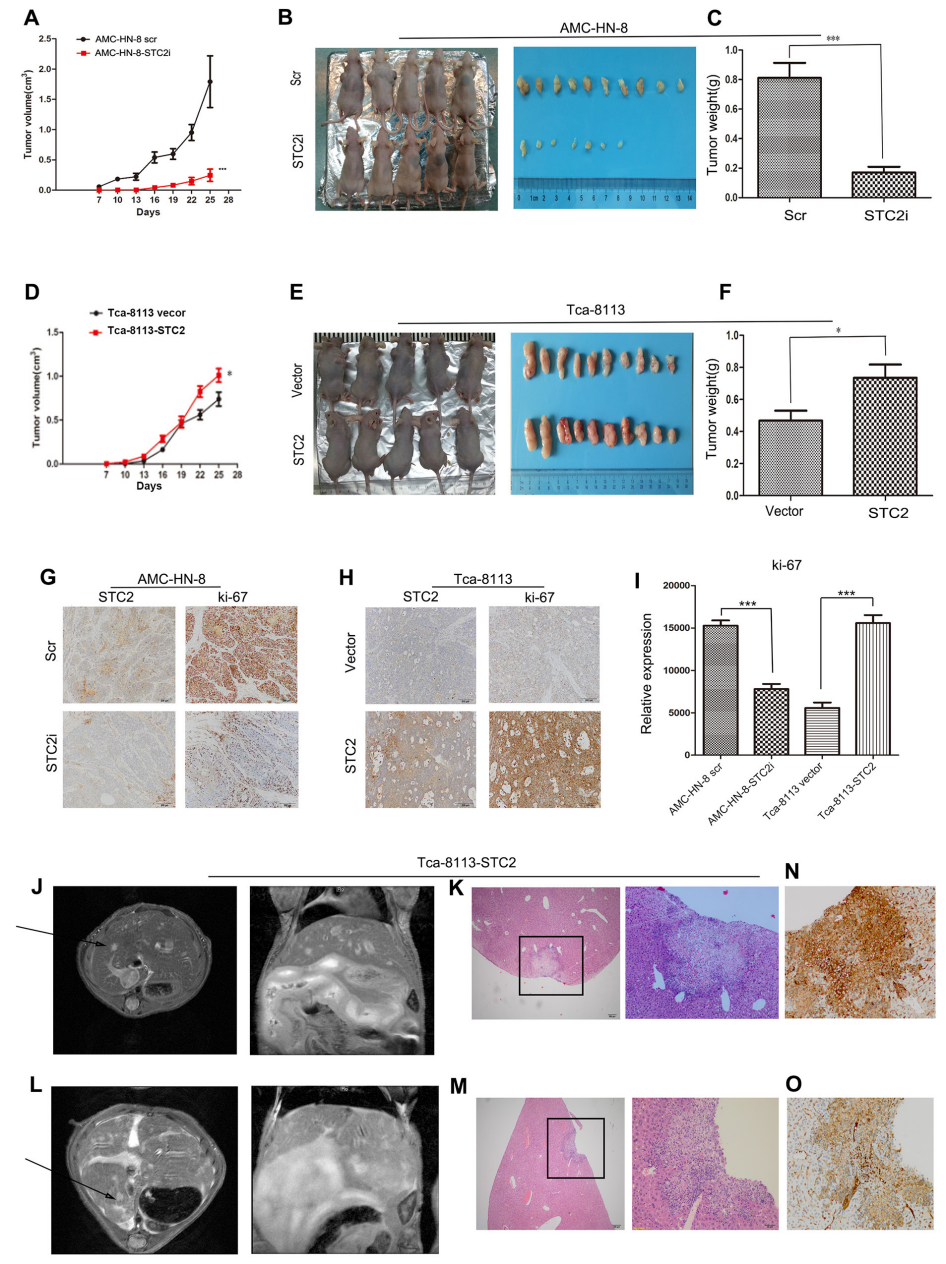
STC2 promotes the growth of HNSCC and tumor metastasis in vivo **A**. *In vivo* tumor growth examined by animal assay. Data are expressed as the mean +/− SD. ***P < 0.001. **B**. Dissection of xenograft tumors. **C**. Quantitative analyses of the weight of the tumor formed in STC2i-animals (***P < 0.001). Error bars = 95% CIs. **D**. *In vivo* tumor growth examined by overexpression of STC2 in animal assay. Data are expressed as the mean +/− SD. *P < 0.05. **E**. Dissection of xenograft tumors. **F**. Quantitative analyses of the weight of the tumor formed in STC2-animals (*P < 0.05). Error bars = 95% CIs. **G, H**. Immunohistochemical analyses of STC2 (G) and Ki67-positive cells (H) in tumor xenograft samples. Magnification, × 200. **I**. The number of Ki67-positive cells per field (five randomly selected visual fields) at 200× magnification. Data are expressed as the mean +/− SD of three samples per group. ***P < 0.001. **J, L**. Selected images from MRI dataset from Tca-8113-STC2 tumour model. Hyper intense lesion in T2-weighted acquisition for both axial plane scanning and coronal scanning are clearly visible (arrowheads). **K, M, N, O**. Metastatic sites were verified with HE staining and p40 staining by immunohistochemistry. Magnification, × 40 and × 200.

To further investigate the effect of STC2 on tumor growth *in vivo*, AMC-HN-8-STC2i-, AMC-HN-8-scr-, Tca-8113-STC2-, and vector-transfected (Vector) cells were injected subcutaneously into one of four groups of nude mice. After 7 days, the neoplasm nodules were visible when the largest diameter measured was > 3 mm. From day 16 to 25 after inoculation, tumor growth was increasingly apparent in the STC2 group compared to the Vector group (*P* < 0.01, Figure 4A, 4D). Specifically, in Figure 4B and 4E, mice injected with cells expressing STC2 cDNA harbored larger tumors compared to animals injected with control cells, whereas animals injected with cells expressing STC2 shRNA developed smaller tumors compared to the control group. In addition, animals in the Scr group developed heavier tumors compared to animals in the STC2i group (Figure 4C, P < 0.001), whereas tumors in the Vector group were lighter than those in the STC2 group (Figure 4F, P <0.05). Furthermore, immunohistochemical staining revealed that the Ki-67 staining index of STC2 shRNA-expressing tumors was reduced compared to that in the Scr group but was enhanced in STC2 overexpressing tumors compared to the vector control (Figure 4G–4I). Together, these data suggest that STC2 promotes HNSCC tumorigenesis *in vivo*.

### STC2 promotes tumor metastasis *in vivo*

Up to 31% of patients with distant metastatic HNSCC presented with liver involvement at the time of death [18]. Liver metastases substantially influence both prognosis and therapeutic options in oncologic patients. Once metastatic tumor cells become established in the liver, they manipulate the blood supply to acquire nutrients and oxygen [19]. Currently, liver metastases are diagnosed using imaging modalities such as magnetic resonance imaging (MRI), computed tomography (CT), positron emission tomography (PET), and ultrasound (US) [20]. To further investigate the effect of STC2 on HNSCC cell invasion, we used MRI to detect liver metastases in our animal model. In the STC2 group, two mice did not present with liver metastases by MRI, while the remaining two animals exhibited hyper intense lesions in T2-weighted acquisition by both axial plane and coronal scanning (Figure 4J, 4L). Neither the four mice injected with vector control cells nor the eight mice injected with parental AMC-HN-8 cells, exhibited signs of metastasis. After MRI scanning, livers were excised from the mice in both the control and metastasis groups and used for histological analysis. As shown in Figure 4K, 4M, 4N, 4O, 4H&4E-stained slides and p40 staining by immunohistochemistry assessed by two pathologists were used to identify metastatic sites. These results suggest that overexpression of STC2 may promote tumor metastasis *in vivo*.

### STC2 modulates HNSCC metastasis through the PI3K/AKT/Snail signaling axis

**Figure 5:**
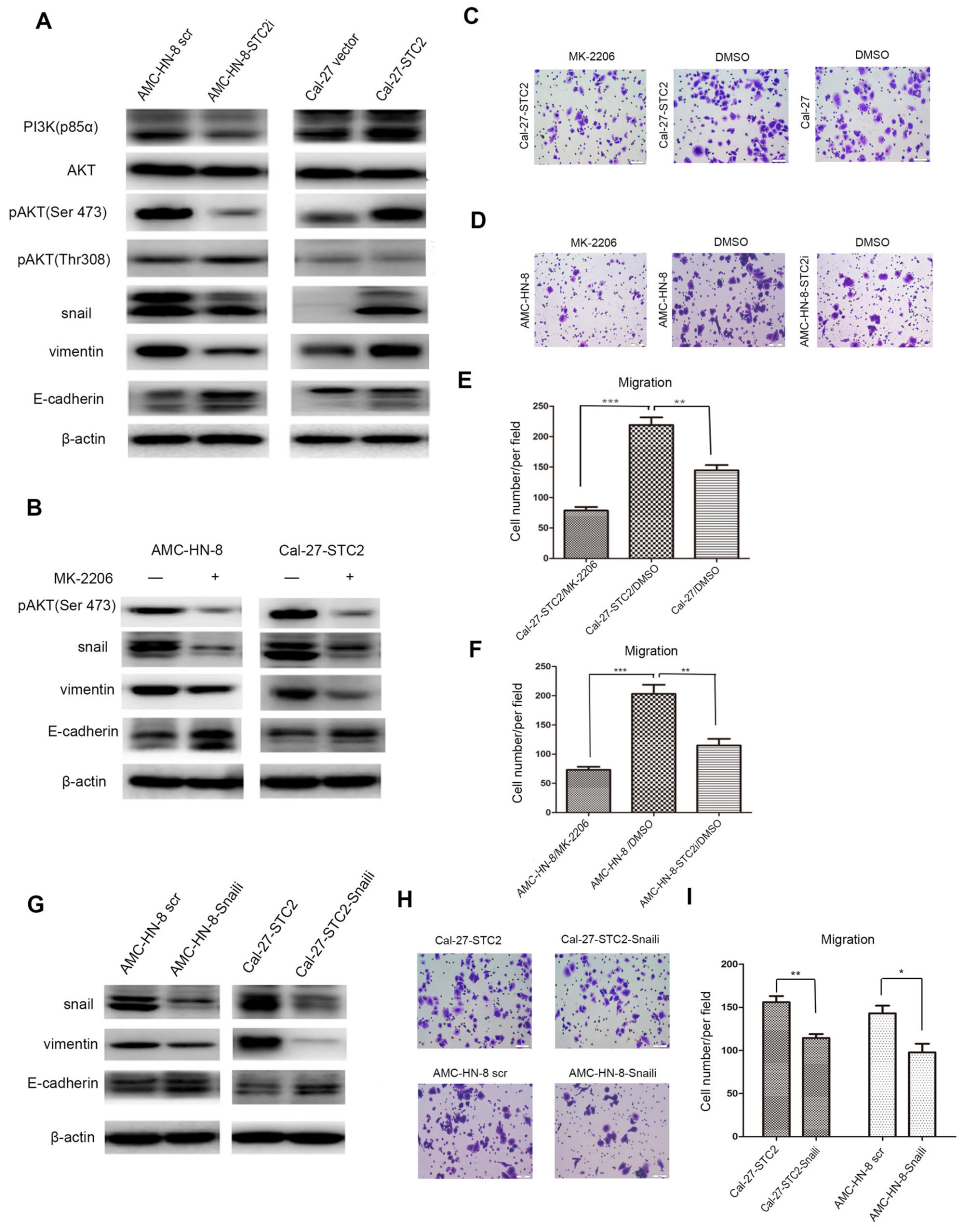
STC2 modulates HNSCC metastasis through the PI3K/AKT/Snail signaling **A**. Detection of molecules associated with PI3K/AKT signaling pathway and EMT program in cell lines by Western blotting. β-actin was used as a loading control. **B**. Detection of pAKT, Snail, Vimentin and E-cadherinin cell lines after treated with1 μM of MK-2206 for 72h. **C-F**. AMC-HN-8 and Cal-27-STC2 cells were treated with DMSO or MK-2206 and then used for migration assay. (**P < 0.01 and ***P < 0.001). **G**. Interruption of Snail expression in AMC-HN-8 scr and Cal-27-STC2 cells altered the expressions of Snail, Vimentin and E-cadherin. **H, I**. Detection of cell migration by using a high throughput screening multi-well insert 24-well two-chamber plates (H). Quantitative analysis of migrated cells (*P< 0.05and **P < 0.01) (I).

The above studies suggest that STC2 promotes metastasis of HNSCC *in vitro* and *in vivo*, but the underlying mechanism is not clear. It is well known that PI3K/AKT signaling is crucial for the sustained survival of cancer cells. In addition, it has also been shown that the PI3K/Akt signaling pathway is activated in HNSCC and plays a crucial role in cell growth and survival [21]. Given that the PI3K/AKT pathway is reported to promote cell proliferation, migration, invasion, and tumor angiogenesis, behaviors that in turn induce tumor metastasis [22], we hypothesized that STC2 might promote HNSCC metastasis through the PI3K/AKT pathway. Thus, we examined the expression of the primary proteins involved in this signaling pathway in both STC2- shRNA and cDNA-treated cells, as well as in their corresponding control cells. Western blot results (Figure 5A) showed that knockdown of STC2 reduced the levels of PI3K-p85α and phosphorylated AKT (Ser473), but not of pAKT (Thr308). In addition, overexpression of STC2 elevated the levels of PI3K-p85α and phosphorylated AKT (Ser473), but did not affect pAKT (Thr308) levels. No significant changes in the levels of total AKT were observed. With respect to proteins associated with metastasis, the expression of Snail was reduced in AMC-HN-8-STC2i cells compared to controls. Moreover, silencing of STC2 decreased levels of vimentin expression, but upregulated the expression of E-cadherin. Conversely, overexpression of STC2 induced the opposite results. These data suggest that STC2 may participate in HNSCC cell motility mainly through PI3K/Akt signaling.

The results presented above suggest that STC2 may inhibit the phosphorylation of AKT at Ser473. To this end, we treated AMC-HN-8 and Cal-27-STC2 cells with the AKT inhibitor MK-2206 to investigate whether the phosphorylation of AKT mediates STC2-induced HNSCC metastasis. We found that treatment of cells with MK-2206 indeed reduced the expression of Snail and vimentin while increasing the expression of E-cadherin (Figure 5B). In addition, a transwell assay revealed that treatment with MK-2206 reduced the migration of Cal-27-STC2 cells compared to Cal-27 cells treated with the vehicle control (DMSO) (Figure 5C, 5E). Moreover, inhibition of pAKT in AMC-HN-8 cells suppressed cell migration compared to controls (Figure 5D, 5F). We then transfected Snail shRNA into AMC-HN-8 and Cal-27-STC2 cells to test whether Snail functions downstream of STC2 and AKT to control STC2-mediated HNSCC metastasis. Silencing of Snail reduced the expression of the metastasis-promoting protein vimentin but enhanced expression of the metastasis inhibitory protein E-cadherin (Figure 5G). Furthermore, knockdown of Snail impaired the migratory ability of AMC-HN-8 and Cal-27-STC2 cells (Figure 5H–5I). Taken together, these results indicate that STC2-induced HNSCC metastasis may be stimulated by PI3K/AKT/Snail signaling.

### Expressions of STC2, pAKT, Snail, vimentin, and E-cadherin in clinical samples

**Figure 6:**
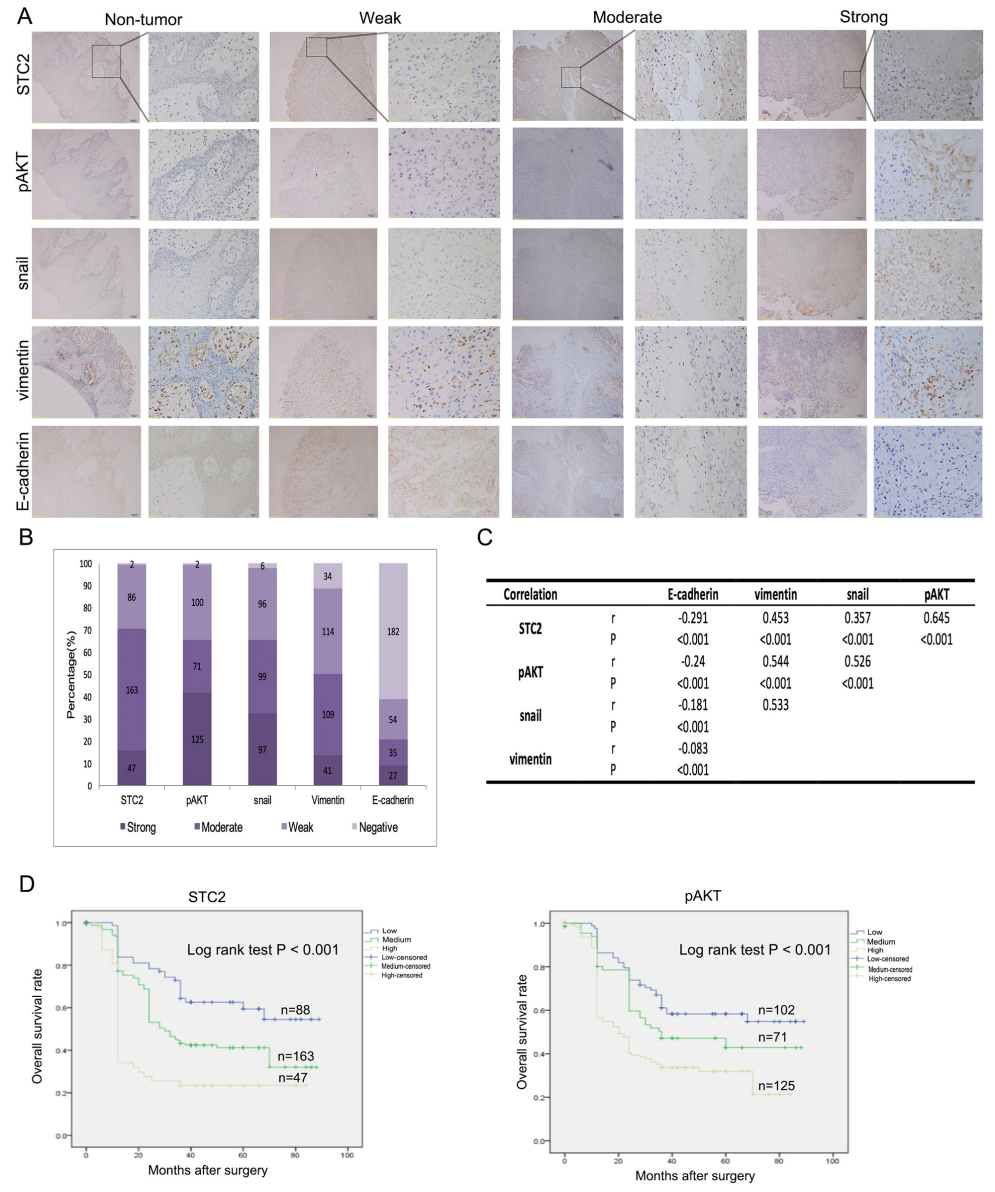
Clinical correlations with expression of STC2, pAKT, Snail, Vimentin and E-cadherin **A**. Representative immunostaining images showing strong, moderate and weak expression of STC2, pAKT, Snail, Vimentin and E-cadherin, respectively. Consecutive slides were stained. Magnification, × 100 and × 400. **B**. Bar graph shows the statistics for the staining intensity of STC2, pAKT, Snail, Vimentin and E-cadherin in tissue arrays containing 289 HNSCC patients. **C**. Statistical correlation of the PI3K/AKT/Snail pathway components in 298 HNSCC patients **D**. Overall survival analysis based on the expression levels of STC2 and pAKT.

As shown above, overexpression of STC2 in HNSCC cells may promote metastasis through the PI3K/AKT/Snail pathway. Therefore, we used immunostaining to detect the expression levels of STC2, pAKT, Snail, vimentin, and E-cadherin in a tissue microarray containing tumor samples from 298 HNSCC patients, as well as tissue from 98 non-tumor controls. We found that E-cadherin was detected in pericarcinous tissues, but STC2, pAKT, Snail, and vimentin were not (Figure 6A). Additionally, compared to non-tumor tissue samples, STC2 expression was high in 15.8% (47/298), moderate in 54.7% (163/298), and weak in 29.5% (86/298) of HNSCC cases (Figure 6B). Likewise, high pAKT, Snail, vimentin, and E-cadherin staining intensities were detected in 41.9% (125/298; moderate, 71/298; weak, 100/298), 32.6% (97/298; moderate, 99/298; weak, 96/298), 13.8% (41/298; moderate, 109/298; weak, 114/298), and 9.0% (27/298; moderate, 35/298; weak, 54/298) of HNSCC cases, respectively (Figure 6B). Given that STC2, pAKT, Snail, vimentin, and E-cadherin are closely associated with HNSCC metastasis, we then explored whether the expression of these markers were interrelated. Notably, using serial tumor sections, statistically significantly positive correlations were found among STC2, pAKT, Snail, and vimentin expression (r = 0.357-0.645, *P* < 0.001, Spearman's correlation) (Figure 6A, 6C), whereas statistically significantly negative correlations were found between E-cadherin and STC2, pAKT, Snail, and vimentin [r= (-0.24)—(-0.083), *P* < 0.001].

An analysis of the associations between STC2 expression and the characteristics of our study population showed that STC2 expression was not significantly associated with patient age, gender, tumor location, or tumor stage. However, STC2 expression was significantly associated with both nodal metastasis and TNM stage (*P* < 0.05) (Table 1). Furthermore, a Kaplan–Meier analysis indicated that high levels of STC2 and pAKT expression led to a significant reduction in overall survival in our cohort of 298 patients (*P* < 0.001) (Figure 6D). Multivariate multivariate statistical analyses demonstrated that STC2 expression was independent factor with prognostic value for OS (*P* =0.012) in patients with HNSCC (Table 2).

**Table 1:** Correlation between STC2 expression and the clinicopathological features in 298 HNSCC specimens

	No. of cases	No. of patients (%)			P-value
		STC2 High	STC2 Medium	STC2 Low	
**Age**					
≤65 years	234	38(16.2%)	127(54.3%)	69(29.5%)	0.91
>65 years	64	9(14.0%)	36(56.3%)	19(29.7%)	
**Gender**					
Male	242	40(16.5%)	131(54.2%)	71(29.3%)	0.756
Female	56	7(12.5%)	32(57.1%)	17(30.4%)	
**Tumor Location**					
Oral cavity	126	15(11.9%)	73(57.9%)	38(30.2%)	0.474
hypopharynx	31	4(12.9%)	18(58.1%)	9(29%)	
larynx	141	28(19.9%)	72(51.0%)	41(29.1%)	
**Tumor Stage**					
T1/T2	209	38(18.2%)	111(53.1%)	60(28.7%)	0.217
T3/T4	89	9(10.1%)	52(58.4%)	28(31.5%)	
**Nodal Metastasis**					
N_0_	122	10(8.2%)	65(53.3%)	47(38.5%)	0.001
≥ N_1_	176	37(21.0%)	98(55.7%)	41(23.3%)	
**TNM Stage**					
I and II	102	18(17.6%)	46(45.1%)	38(37.3%)	0.018
III and IVa	89	15(16.9%)	59(66.2%)	15(16.9%)	
IVb and IVc	107	14(13.1%)	58(54.2%)	35(32.7%)	
**Pathological differentiation**					
Moderately and highly differentiated	242	39(16.1%)	133(55.0%)	70(28.9%)	0.873
Poorly differentiated	56	8(14.3%)	30(53.6%)	18(32.1%)	
**Smoking index**					
<600	144	22(15.3%)	76(52.8%)	46(31.9%)	0.677
≥600	154	25(16.2%)	87(56.5%)	42(27.3%)	
**Drinking**					
None or occasionally	146	23(15.8%)	76(52.0%)	47(32.2%)	0.591
Frequently	152	24(15.8%)	87(57.2%)	41(27.0%)	

**Table 2:** Results of multivariate survival analyses for overall survival

Multivariate survival analysis	Categories	HR(95%CI)	P value
**STC2**			0.024
	Median	0.405(0.227 - 0.723)	0.002
	Low	0.612(0.393 - 0.953)	0.030
**Nodal Metastasis**	No / Yes	1.895 (1.079 - 3.329)	0.026
**TNM Stage**	I and II / III and IVa / IVb and IVc	1.043(0.745 - 1.460)	0.806
**Invasion**	No / Yes	0.841(0.602 - 1.176)	0.311
**Pathological differentiation**	Moderately and highly / poorly differentiated	1.029(0.672 - 1.574)	0.897

## DISCUSSION

To the best of our knowledge, this is the first comprehensive study to provide evidence that STC2 increases the growth of HNSCC, STC2 is a positive regulator of HNSCC metastasis, and STC2-mediated HNSCC metastasis may be regulated by the PI3K/AKT/Snail pathway.

Stanniocalcin (STC) is a glycoprotein hormone that was originally discovered in the corpuscles of Stannius, an endocrine gland in fish [23, 24]. STC2, a member of the STC family of molecules, is thought to modulate calcium and phosphate homeostasis [25, 26]. As reported by many studies, the effects of STC2 on cell proliferation are controversial. For example, Law et al. reported that overexpression of STC2 in SKOV3 cells stimulated proliferation under hypoxic conditions [11]. However, Raulic et al. showed that overexpression of STC2 significantly impaired the growth of breast cancer cells [16]. Moreover, Ito et al. reported that overexpression of STC2 selectively protected HeLa cells from endoplasmic reticulum stress-induced cell death [6]. These controversial results may indicate that the responses of STC2 may differ depending on the presence of specific stimuli and may be influenced by different signaling pathways. We therefore sought to investigate the function of STC2 in the development of HNSCC. In this study, we show that STC2 promotes HNSCC cell proliferation and suppresses cell apoptosis *in vitro*. In addition, according to previous studies, the G1/S cell cycle checkpoint is frequently dysregulated in tumor cells. Accordingly, in our study, flow cytometric analysis revealed that STC2 expression promotes passage through the G1/S cell cycle transition.

Previous studies have demonstrated that overexpression of STC2 increases cancer cell migration and motility [11, 17] and is correlated with metastatic spread in cancer tissue. However, Raulic et al. also showed that loss of STC2 was positively correlated with aggressive phenotypes in breast cancer [16]. In our study, we found that high expression of STC2 promotes the migration and invasion of HNSCC cells *in vitro* and *in vivo*. In addition, an analysis of 298 HNSCC tissue samples demonstrated that STC2 was upregulated in over two-thirds of human HNSCC cases and was significantly associated with nodal metastasis and TNM stages. Moreover, STC2 overexpression in HNSCC was strongly correlated with reduced overall survival, consistent with reports that STC2 is a poor prognostic marker for patients with renal cell carcinoma and gastric cancer [27, 28].

Although HNSCC is heterogeneous in nature, alterations in major components of the PI3K/Akt pathway are constantly observed throughout the development of most HNSCC cases. These alterations include genetic aberrations, such as mutations or DNA copy number amplification, and dysregulation of mRNA or protein expression. Protein kinase B (AKT) is an effector protein downstream of phosphoinositide 3-kinase (PI3K) and plays important roles in carcinogenesis [29]. We found that silencing of STC2 significantly reduced levels of pAKT (Ser473) and total AKT but had no effects on pAKT (Thr308) levels. Moreover, activation of the PI3K/AKT/Snail signaling pathway has been reported during EMT in the colon [30]. Epithelial-mesenchymal transition (EMT), a process by which cancer cells lose their epithelial properties and acquire mesenchymal phenotypes such as motility and invasiveness [31–33], is activated synergistically by a number of factors including Slug, Snail, E-cadherin, Twist, and vimentin [34–39]. Snail is a well-described zinc finger (ZF) transcriptional repressor that is essential for embryonic development [40]. However, given its role in promoting lymphovascular invasion and regional metastasis, other studies have shown that Snail may also be used as a molecular prognostic marker for HNSCC [41, 42]. Although Hou et al. reported that STC2 may inhibit EMT in human breast cancer cells [43], our results suggest that the interaction between Snail and the PI3K/AKT pathway may mediate STC2-induced HNSCC metastasis. Similarly, the expression patterns of STC2, pAKT, Snail, vimentin, and E-cadherin in HNSCC tissues were consistent with those in cells, implying that STC2 plays an oncogenic role in HNSCC.

In summary, the results presented herein demonstrate that STC2 promotes HNSCC cell proliferation, tumor growth, and metastasis through the PI3K/AKT/Snail pathway. These data suggest that STC2 may be a potential marker and therapeutic target for the diagnosis, prognosis, and treatment of human HNSCC.

## MATERIALS AND METHODS

### Cell lines and cell culture

Human pharynx squamous cell carcinoma cells (FaDu) and 293T lentiviral packaging cells were purchased from the American Type Culture Collection (Manassas, VA). Human laryngeal squamous cell carcinoma cells (AMC-HN-8) were obtained from the Eye, Ear, Nose, and Throat Hospital of Fudan University, whereas human tongue squamous cell carcinoma cell lines (Tca-8113 and Cal-27) were obtained from the Ninth People's Hospital, Shanghai Jiao Tong University School of Medicine. Cells were maintained in the recommended medium supplemented with 10% fetal bovine serum, 100 U/ml penicillin, and 100 μg/mL streptomycin. The cultures were incubated at 37°C in a humidified 5% CO_2_ atmosphere.

### Cloning of and viral infection with STC2 shRNA, Snail shRNA, and STC2 cDNA

A small hairpin RNA (shRNA) targeting 5’-gaatgctacctcaagcacga-3’ (NM_003714.2, position 412-431) of STC2 mRNA was cloned into the pBabe-U6-puromycin retroviral vector (herein referred to as STC2i) and delivered to AMC-HN-8 and FaDu cells as previously described [44]. The oligonucleotide sequences used to generate a shRNA targeting the open reading frame of Snail mRNA were 5′-TGCACATCCGAAGCCACAC-3′. Retroviruses carrying the Snail shRNA were used to infect AMC-HN-8 and Cal-27-STC2 cells. The oligonucleotide sequence used to generate a scrambled shRNA was 5´-CTAGCGGTATCGTGTGAGT-3´. Control cell lines were generated by infection with viruses containing the scrambled shRNA following the same protocol. The retroviral infection was performed using a previously published method [45]. After infection and selection with STC2i or the scrambled shRNA, the resulting cell lines were named AMC-HN-8-STC2i or FaDu-STC2i, and AMC-HN-8 scr or FaDu scr, respectively. Cells were selected with puromycin (2.0 μg/mL) for 10–14 days.

The coding DNA sequence of STC2 were inserted into the overexpressing plasmid pLenti6/V5 lentivirus vector. The control vector used in this study was a pLenti6/V5-LacZ lentivirus vector (Invitrogen, USA). These plasmids were transfected into 293T packaging cells to generate lentiviruses, which were then used to infect target cell lines according to previously described methods [45]. Lentiviruses carrying STC2 cDNA were used to infect Tca-8113 and Cal-27 cells.

### Western blotting

Western blotting was carried out according to standard methods. Briefly, total protein extracts were obtained by lysing the cells with RIPA buffer containing a protease inhibitor cocktail (BestBio, Shanghai, China). Protein concentrations were then determined for all samples using the BCA protein assay (BioTech Well, Shanghai, China). Equal volumes of each sample were loaded onto a 10% polyacrylamide gel, separated by SDS–polyacrylamide gel electrophoresis, and transferred onto a PVDF membrane (Millipore, Billerica, MA, USA). After blocking with 5% non-fat dry milk, the membrane was incubated with the corresponding primary and secondary antibodies, and immunoreactive bands were visualized by enhanced chemiluminescence (ECL) (Millipore, Billerica, MA, USA). An antibody against STC2 (60063-1-Ig) was purchased from Proteintech and antibody Snail (ab180714) was obtained from Abcam. Antibodies against Ki67 (sc-23900), pAkt (sc-7985-R), vimentin (sc-73258), and β-actin (sc-47778) were purchased from Santa Cruz Biotechnology. Antibodies against PI3k (cs-4292), AKT (cs-4691s), E-cadherin (cs-14472S) were obtained from Cell Signaling Technology. All experiments were repeated three times.

### Cell proliferation assay

Approximately 1×10^3^ cells suspended in 100 μl of medium were plated in duplicate wells of 96-well plates. Following an overnight incubation to facilitate attachment, the cell culture medium was refreshed, and cell proliferation indices were assessed daily using the Cell Counting Kit-8 (CCK-8, Dojindo, Molecular Technologies) according to the manufacturer's instructions. Growth curves were then generated using the OD values obtained from the CCK-8 assay.

### Colony formation assay

To test colony formation, cells were seeded into 6-well plates (500 cells per well in triplicate) and cultured in DMEM supplemented with 10% FBS. After a 12-day incubation period, the cells were washed and fixed with 4% paraformaldehyde and stained with crystal violet. Colonies with more than 50 cells were counted.

### Annexin V/7AAD staining

Flow cytometry was performed to examine the percentage of cells actively undergoing apoptosis with Annexin V-PE/7AAD Apoptosis Detection Kit (Roche, Shanghai, China). 1 × 10^6^ cells were harvested and washed with PBS. Cells were mixed with 5 μl Annexin-V-PE and 10 μl 7AAD and then resuspended in 500 μl binding buffer. Flow cytometric analysis (FC500 MPL, Beckman Coulter, CA, USA) was performed immediately after supravital staining. The cells in early stages of apoptosis were Annexin V positive and 7AAD negative, whereas the cells in the late stage of apoptosis were both Annexin V and 7AAD positive.

### Cell cycle analysis

Fluorescence-activated cell sorting (FACS)-based analysis of DNA content was used to evaluate the cell cycle distributions of our retrovirally infected cells. Cells were harvested as single cell suspensions, washed with cold PBS, and fixed in 70% ethanol overnight at -20°C. The single-cell suspensions were then centrifuged at 1500 r/min for 5 minutes, re-suspended in 1 mg/ml RNase solution for 30 min at RT, and incubated with 0.1 mg/ml PI (DNA-PrepTM Reagents Kit, CA) at 4°C for 1 hour in the dark. Samples were analyzed by flow cytometry (Beckman, USA).

### Wound-healing assay

To evaluate cell motility, a wound healing assay was performed. Cells (approximately 4×10^5^/well) were seeded in 6-well culture plates containing DMEM with 10% FBS. After 24 h, confluent cells were scratched with a 10-µl pipette tip, and wounded monolayers were then washed three times with PBS to remove cell debris. After a 48-h incubation period, cells that had migrated into the wounded area or that were protruding from the border of the wound were visualized and photographed using an inverted microscope. Each experiment was performed at least three times independently.

### Migration and matrigel invasion assay

For the cell migration assay, a 24-well plate containing 8-μm (pore size) polycarbonate membrane inserts (BD Biosciences, San Jose, CA) was used. Approximately 4×10^4^ cells were added to the upper chamber of each well and allowed to migrate at 37°C for 24-36 hours toward the lower reservoir, which contained culture medium with 2.5% fetal bovine serum. Cell invasion was tested using the Transwell chamber invasion assay (Matrigel-coated membrane, BD Biosciences, Bedford, MA). 1.0×10^4^ cells were seeded in serum-free medium into the upper chamber and allowed to invade toward 15% fetal calf serum as a chemoattractant in the lower chamber. After 24-48 h (according to their respective invasive ability), cells that had invaded through the Matrigel matrix and adhered to the underside of the membrane were fixed in 4% paraformaldehyde for 15 minutes and stained with crystal violet for 30 minutes. All cells were counted at ×200 magnification under a microscope. The assay was repeated three times with duplicate samples, and five fields were counted for each sample.

### Chemicals

MK-2206, a compound that inhibits auto-phosphorylation of AKT at position Ser473 and Thr308, was purchased from Selleck and dissolved in DMSO. Throughout the study, the final concentration of DMSO in our cell cultures did not exceed 0.1%. Cells were grown to 60–70% confluency, treated with 1 μM MK-2206 for 72 h, and digested with trypsin for use in the following experiments.

### Establishment of xenograft tumor models

All animal protocols were approved by the Institutional Animal Ethical Committee of Fudan University, China, and adequate measures were taken to minimize animal pain and discomfort. Twenty 5-week-old female BALB/C nude mice were randomly divided into four groups and bred under specific pathogen-free (SPF) conditions in a temperature- (25±2°C) and humidity- (50±5%) controlled facility on a 12 h light, 12 h dark cycle. Log phase AMC-HN-8 scr, AMC-HN-8-STC2i (3×10^6^), Tca8113-empty vector, or Tca8113-STC2 cDNA (3×10^6^) cells were re-suspended in 0.2 ml of PBS and injected into the subcutaneous tissue around the neck. The length and width of the tumors (in mm) were measured weekly using calipers, and the mice were sacrificed when the tumors reached 1.5 cm in diameter. The tumors were excised and weighed, and the tumor volume was calculated using the formula (a×b^2^) ×0.5, where a and b indicate the long and short dimensions, respectively.

For the tail-vein injection group, 16 mice were randomly divided into four groups (four mice per cell line) and each mouse received one injection of 2 × 10^6^ cells. Mice were monitored for weight loss, lethargy, and poor appetite and were sacrificed timely before natural death occurred. Animal assays were repeated twice.

### *In vivo* magnetic resonance imaging

MRI was approved and performed by the Department of Nuclear Medicine, Fudan University Shanghai Cancer Center. Data were acquired using a 7-T horizontal bore magnet (Bruker Biospec 70/20USR, Ettlingen, Germany). A transmitter-receiver quadrature volume coil with an inner diameter of 38 mm was used for collection of MR data. For MR scans, animals were anesthetized by inhalation of a mixture of oxygen and 4% isoflurane. This anesthesia condition was maintained throughout the experiments. The mice were placed inside the coil in the prone position, positioned in the scanner bed with the imaging field of view (FOV) centered at the abdomen, and kept warm with a pad with a continuous warm water supply. The T2-weighted acquisition for axial plane scanning was performed with TE = 33.00 ms; TR = 2500.000 ms; slice thickness 0.700 mm; image size 256 × 256; field of view (FOV) = 30 × 25 mm, thus giving an isotropic resolution of 117 × 98 μm with four signals acquired. T2-weighted acquisition for coronal scanning was performed with TE = 15.27 ms; TR = 1365.041 ms; slice thickness 0.800 mm; image size 256 × 256; field of view (FOV) = 30 × 28 mm, thus giving an isotropic resolution of 117 × 109 μm with four signals acquired.

### H&E staining and immunohistochemistry

Paraffin-embedded tissues were sectioned for H&E staining and immunohistochemical analysis. For H&E staining, slides were deparaffinized, hydrated, and stained with hematoxylin for 1 min. After rinsing, the slides were stained with eosin for 1 min, rinsed, and sealed with cover slips using Permount TM Mounting Medium. For immunohistochemistry (IHC), samples were fixed in 10% formalin and embedded in paraffin wax. Then, 3-mm sections were cut from the paraffin blocks for IHC analysis. The sections were stained with mouse anti-Ki67 (1:500), mouse anti-STC2 (1:1000) and p40 (prediluted, Biocare Medical) at 4°C overnight. Ki67-positive and p40-positive cells were identified according to the instructions specified in the immunohistochemistry kit. The average number of Ki67-positive cells in one tumor sample was calculated.

### Patient information and immunohistochemical staining

The use of tissue blocks and access to patient medical records were approved by the Institutional Review Board of Fudan University Shanghai Cancer Center. Paraffin-embedded tissue samples were selected from 298 patients who were diagnosed with HNSCC and received surgery at Fudan University Shanghai Cancer Center between January 2007 and December 2012. Patients selected for inclusion in this study were diagnosed with primary squamous cell carcinoma of the oral cavity, oropharynx, pharynx, or larynx without other malignancies and no prior history of radiotherapy or chemotherapy. In addition, patient clinical and pathological data were collected including age, anatomic tumor location, treatment, differentiation grade, lymph node metastasis, recurrence, and survival time. Patient follow-up and postoperative management data were collected by telephone or obtained from outpatient records. The median follow-up duration of all patients was 28.0 months (ranging from 2.0 to 89.0 months). At the last follow-up, 118 patients (39.6%) were alive, 157 (52.7%) were dead, and 23 (7.7%) were lost to follow-up. Tumor stage (T-stage) was classified based on the 2002 TNM staging system of the Union for International Cancer Control (UICC). Tissue microarrays consisting of tumor samples from the 298 HNSCC patients, as well as 98 cancer-adjacent normal tissue samples, were constructed with an arraying machine (Beecher Instruments). For each case, two replicate 1-mm-diameter cores were collected, and each was sliced into 4-mm-thick sections for immunohistochemical (IHC) analysis. The expression of STC2, pAKT, Snail, vimentin, and E-cadherin was detected by immunohistochemical staining. The antibodies used to detect STC2, pAKT, Snail, vimentin, and E-cadherin were described above. Paraffin-embedded tissue sections were pre-treated and stained with antibodies using a previously reported method [46, 47]. The secondary antibodies against mouse or rabbit IgG were obtained from an IHC kit (#CW2069, Beijing CoWin Bioscience Co. Ltd, Beijing, China). Staining was independently assessed and scored in a blinded fashion by two pathologists (Drs. Bin Chang and Fangfang Zhong). The staining scores for STC2, pAKT, Snail, vimentin, and E-cadherin were determined based on both the percentage and intensity of positively stained cells. An unequivocal positive reaction was defined as a brown signal in the cytoplasm or on the cell membrane. To estimate associations between IHC staining and clinical variables, the percentage of positive cells (PPC) was classified as follows: 0 = ≤ 5% of positive cells, 1 = 5 - 25%, 2 = 25-50%, 3 = 50 -75%, and 4 = ≥ 75% of positive cells. The intensity of staining (IS) was classified as 0, negative (−); 1-3, weakly positive (+); 4-6, moderately positive (++); and > 6, strongly positive (+++). As for the negative control, the primary antibody was replaced with antibody diluent. The final protein expression score was calculated with the formula PPC × IS and was considered “low” for 0 - 4, “medium” for 4 - 8, and “high” for 8 - 12.

### Statistical analysis

Statistical analyses were performed with GraphPad Prism 5 and SPSS version 18.0 for Windows (IBM). Group comparisons of normally distributed data were performed using t-tests (for two samples) or one-way ANOVA (for multiple comparisons). In addition, the non-parametric Wilcoxon test was used to analyze continuous data that did not follow a normal distribution, and the Tukey-Kramer test was applied following ANOVA for multiple comparisons. Spearman's correlation coefficients, and the Mann-Whitney U test were used where appropriate. Categorical variables were compared using a χ^2^ analysis or Fisher's exact test. The relationships between STC2 or pAKT and overall survival were determined by Kaplan-Meier analysis (log-rank test). Multivariate analyses were performed with the Cox proportional hazards model. *P* < 0.05 (two-tailed) was considered statistically significant.
